# Congenital partial arhinia: a case report

**DOI:** 10.1186/1752-1947-1-97

**Published:** 2007-09-20

**Authors:** Guzin Akkuzu, Babur Akkuzu, Erdinc Aydin, Murat Derbent, Levent Ozluoglu

**Affiliations:** 1 Department of Otolaryngology, Başkent University, Ankara, Turkey; 2 Department of Pediatrics, Başkent University, Ankara, Turkey

## Abstract

Congenital arhinia is an extremely rare anomaly consisting of an absence of external nasal structures and nasal passages. Fewer than 30 cases have been reported. Patients with a familial absence of the nose have been reported, but the effects of genetic and maternal factors are unknown. Midface hypoplasia may accompany arhinia. Accompanying malformations are thought to be caused by an absent or rudimentary nose. A patient with partial congenital arhinia is presented and the embryology and literature review are discussed.

## Background

Congenital absence of the nose, *arhinia*, is an extremely rare anomaly. Arhinia may be accompanied by midline defects, or ear, palatal, ocular, or facial abnormalities. Severe airway and feeding problems may accompany arhinia in newborns.

## Case presentation

**Figure 1:**
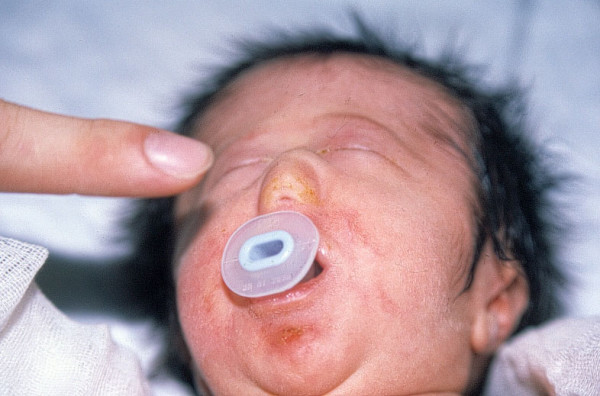
Photograph of the patient.

**Figure 2:**
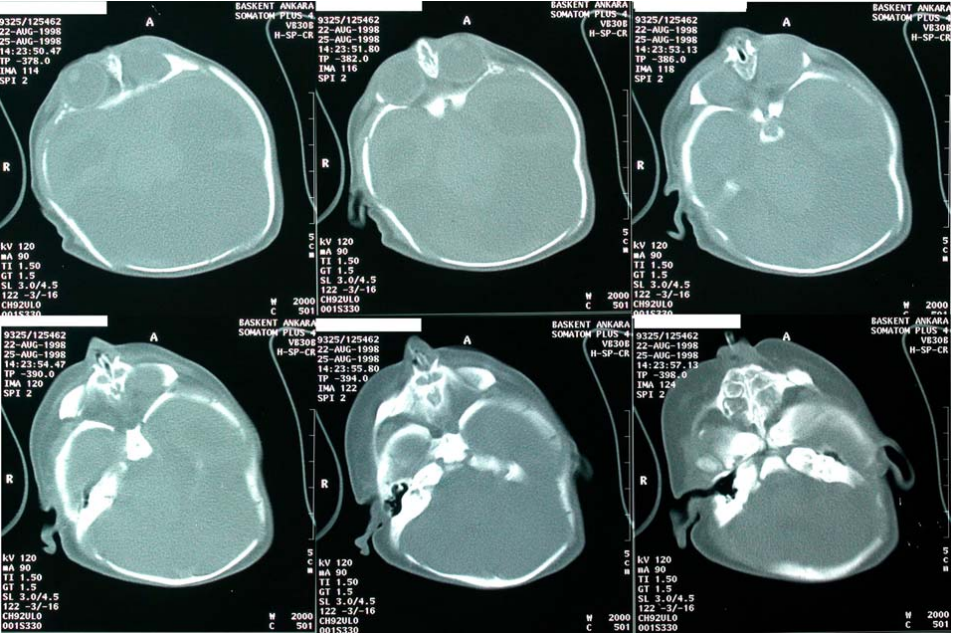
Consecutive axial computed tomography sections from superior to inferior demonstrating absence of bony nasal structures and paranasal sinuses. A catheter is placed inside the single-blind cavity demonstrating a lack of communication with the pharynx.

Our case is a female patient, born to a 28-year-old mother. This was the mother's first pregnancy. The mother denied any antenatal problems, and the baby was born vaginally at full term. Family history revealed no interrelative marriage or history of congenital malformation. Results of a chromosomal analysis showed a normal female. The baby was born with severe nasal deformation. On gross inspection the external nose was absent. The nasal dorsum structures and remnants of the alar cartilages were barely palpable. The columella and right nostril were absent; the left nostril was severely stenotic (Figure 1). Probing and dilating revealed a small cul-de-sac. Submucosal cleft palate, right auricular deformity, and hypotelorism were also present. Axial computerized tomography showed that the nasal and paranasal structures were not developed (Figure 2). A cul-de sac was present in place of the intranasal structures. Intracranial midline structures such as the septum pellucidum, falx cerebri, corpus callosum, and third ventricle were absent. The lateral ventricles and the third ventricle were replaced by a monoventricle. Cerebral hemispherical cortical sulci and a sylvian fissure were not observed. A 7-cm posterior intracranial cyst was present. These findings were consistent with alobar holoprosencephaly. The family declined any surgical intervention such as tracheotomy, and the patient was discharged with an oral airway.

## Discussion

During facial development, cranial neural crest cells migrate from the trigeminal nerve region to the face [1]. Development of the nose and nasal cavities occurs between the third and tenth weeks of gestation [1]. Nasal placeoda appear as local thickening of the surface of the ectoderm and develop from the frontal process advancing laterally between the medial and lateral nasal processes. The nasal placeodas invaginate at the fifth week to form the nasal nuclei. Nostrils develop from the nasal nuclei. The nasal nuclei migrate posteriorly to form nasal cavities. Meanwhile, the oral and nasal cavities are separated by bucconasal membranes that will rupture at the seventh or eighth weeks to form the posterior nares. The nasal septum develops at the ninth week when the palate and inferior septum unite and form the secondary palate. Hard palate development finishes at the eighth or ninth week, and the soft palate finishes at the 11th or 12th weeks.

The pathogenesis of arhinia is not clearly understood. The proposed mechanism may be a developmental defect in the medial and lateral nasal processes or overdevelopment and early fusion in the medial nasal processes [2]. Arrest of absorption of the nasal epithelial plates at the 13th through the 15th week may be another possible mechanism. Abnormal migration of neural crest epithelial cells is another possible explanation.

Olsen and associates reviewed the literature through 2001 and collected 22 additional cases [3]. McGlone and associates collected 27 cases until 2003 and investigated the common abnormalities [4]. As was true in our case, most of the collected cases had had an uneventful antenatal history.

Congenital arhinia is a very rare defect during embryogenesis. Most cases are sporadic but familial cases have been reported. Mostly the karyotype is normal. In 2 cases, anomalies in chromosome 9-and in a single case, chromosome 3–12 translocation – have been detected [5]. A variety of anomalies such as absence of the paranasal sinuses, cleft palate, facial anomalies like hypotelorism or hypertelorism, central nervous system anomalies, umbilical hernia, syndactylia, and hypospadias may accompany the arhinia [3,4]. Computed tomography and magnetic resonance imaging are helpful in detecting accompanying anomalies and planning further surgical treatment.

We considered our patient as having partial arhinia because there were some remnants of the external nose and a cul-de-sac was present. This was accompanied by central nervous system and ear anomalies, a submucosal cleft palate, and hypotelorism.

## Conclusion

Congenital arhinia is an extremely rare condition of unknown etiology. Facial anomalies and other concomitant distant anomalies may be present. These patients experience serious problems with regard to an open airway and feeding. Any accompanying anomalies should be detected and surgical correction should be planned.

## Consent

Written informed consent was obtained from the patient's parents for publication of this case report and any accompanying images. A copy of the written consent is available for review by the Editor-in-Chief of this journal.

## Competing interests

The author(s) declare that they have no competing interests.

## Authors' contributions

GA made substantial contributions with regard to the manuscript's conception and design and was involved in drafting the manuscript; the author will give final approval of the version to be published. BA made substantial contributions with regard to the manuscript's conception and design and was involved in drafting the manuscript; the author will give final approval of the version to be published. EA made substantial contributions with regard to the manuscript's conception and design and was involved in drafting the manuscript; the author will give final approval of the version to be published. MD made substantial contributions with regard to the manuscript's conception and design and was involved in drafting the embryologic part of the manuscript. LO was involved in revising the manuscript critically; the author will give final approval of the version to be published.
